# Cut-off values of serum homocysteine and high-sensitivity CRP for predicting early neurological improvement after intravenous thrombolysis

**DOI:** 10.1186/s12883-026-05064-4

**Published:** 2026-06-17

**Authors:** Mi-Suk Oh, Kwang-Yeol Park, Hae-Bong Jeong, Soo Hyun Cho, Chan-Young Park

**Affiliations:** 1 Department of Family Medicine, Chung-Ang University Hospital, 102 Heukseok-Ro, Dongjak-Gu, Seoul, 06973 Republic of Korea; 2 Department of Neurology, Chung-Ang University Hospital, 102 Heukseok-Ro, Dongjak-Gu, Seoul, 06973 Republic of Korea

**Keywords:** Homocysteine, High-sensitivity C-reactive protein, Large artery atherosclerosis, Early neurological improvement, Recombinant tissue plasminogen activator

## Abstract

**Background:**

We aimed to determine cut-off values of factors affecting early neurological improvement in patients after recombinant tissue plasminogen activator therapy for acute ischemic stroke.

**Methods:**

A total of 135 patients with acute ischemic stroke treated with tissue plasminogen activator within 4.5 h of stroke onset were enrolled at a single center. Therapy outcomes were based on early neurological improvement, defined as when the NIHSS score after 24 h of tPA treatment decreased by ≥ 40% from the baseline value. Binary logistic regression analysis was used to identify the factors affecting early neurological improvement.

**Results:**

Sixty-nine (51.1%) patients had early neurological improvement. Multivariate analysis showed that younger age, absence of diabetes mellitus, presence of hypertension, lower serum homocysteine (odds ratio, 0.865; 95% confidence interval, 0.787–0.950; *P = *0.003) and lower high-sensitivity C-reactive protein (0.345; 0.203–0.586; *P < *0.001) levels were significantly associated with a higher probability of early neurological improvement. The cut-off values of serum homocysteine and high-sensitivity C-reactive protein were 11.99umol/L and 1.16mg/L, respectively. If Hcy and hsCRP were combined and used together, it was possible to predict ENI better than when Hcy and hsCRP was used alone. (sensitivity 92.75, specificity 60.61, and optimal cut-off value 23.65) ROC analyses showed modest discrimination for homocysteine (AUC 0.743, 95% CI 0.660–0.814) and hsCRP (AUC 0.747, 95% CI 0.665–0.818), with improved discrimination when both biomarkers were combined (AUC 0.797, 95% CI 0.719–0.861).

**Conclusions:**

In IVT-treated AIS, lower homocysteine and hsCRP were independently associated with early neurological improvement.

## Highlights

Early neurological improvement is defined as when the NIHSS score after 24 hours of tPA treatment decreased by ≥ 40% from the baseline value.

Significant factors affecting early neurological improvement were serum homocysteine and high-sensitivity CRP levels.

Cut-off values of serum homocysteine and high-sensitivity CRP levels affecting early neurological improvement after intravenous thrombolysis were 11.99umol/L and 1.16mg/L. If Hcy and hsCRP were combined and used together, it was possible to predict ENI better than when Hcy and hsCRP was used alone.

## Introduction

Acute ischemic stroke (AIS) is the second leading cause of death worldwide, with increasing mortality rates [1].

In Korea, in 2020, the prevalence of stroke among adults over 19 years of age was 1.71%, with approximately 105,000 cases occurring every year. Of these, 76% were ischemic stroke cases, and the remaining 24% were cases of stroke resulting from intracerebral or subarachnoid hemorrhage [2].

In patients with AIS due to large vessel occlusion, recanalization of the occluded vessel is the most important factor affecting the prognosis [3]. Intravenous recombinant tissue plasminogen activator (tPA), also known as alteplase, is a glycoprotein that directly activates and induces plasminogen-converted plasmin. This results in fibrin degradation and thrombolysis [4]. tPA is used for primary reperfusion therapy and is the most economical and convenient treatment method.

Early neurological improvement (ENI) assessment has been used as a 24-h measurement to assess changes in neurological function [5]. There have been attempts to use ENI as a treatment outcome of AIS patients and it also indicates a good prognosis [6, 7]. However, researchers have not applied consistent definitions of ENI [8]. Therefore, the purpose of the present study was to find out optimal ENI and factors affecting ENI.

Homocysteine and high-sensitivity C-reactive protein are biomarkers reflecting vascular inflammation and atherosclerotic burden, both of which are closely related to ischemic stroke pathophysiology and clinical outcomes. Furthermore, we aimed to get cut-off values of the factors which are associated with ENI.

## Methods

### Thrombolysis methods

Alteplase was used because it is the only thrombolytic agent approved by the United States Food and Drug Administration for AIS [9]. The recommended alteplase was 0.9 mg/kg. Initially, 10% of the total treatment dose was administered as a bolus for 1 min. The remaining 90% was dissolved in normal saline and infused intravenously for 60 min. Blood samples for Hcy and hsCRP measurements were obtained at hospital admission, approximately 1–2 h after initiation of intravenous thrombolysis, as part of routine laboratory evaluation. Given the pharmacokinetic profiles of both biomarkers, their levels are not expected to be acutely altered by tPA administration within this timeframe.

### Definition of early neurological improvement

Patients were evaluated based on the differences between baseline NIHSS and NIHSS scores after 24 h of IVT. The results are expressed as the ENI. Researchers have commonly defined ENI as a reduction in the NIHSS score by ≥ 4 [10]. However, some studies suggested that percent change improvement in NIHSS may be a better marker of thrombolytic activity [11, 12]. In addition, some studies defined ENI using various percentage values and found that the best clinical outcome could be predicted when the NIHSS score improved by more than 40% within 24 h after treatment [13, 14]. Therefore, we defined ENI as when the NIHSS score after 24 h of tPA treatment decreased by ≥ 40% from the baseline value in order to apply more accurate standards.

### Statistical analysis

Odds ratios (ORs) and their corresponding 95% confidence intervals (CIs) were calculated using SPSS™ statistics v25.0(IBM, Armonk, NY, USA).

Quantitative data were presented as mean ± standard deviation values, while categorical variables were expressed as absolute values (percentages). The chi-square test was used to compare patients with favorable and unfavorable outcomes as categorical variables. The Student’s t-test was used to compare groups with normally distributed quantitative variables.

Binary logistic regression models were used to evaluate the association between serum homocysteine (Hcy) and high-sensitive C-reactive protein (hsCRP) levels and stroke outcomes according to NIHSS scores. Candidate variables for regression analyses were predefined based on previously reported predictors of stroke outcomes and routinely collected clinical variables in the stroke registry. Variables with *P* ≤ 0.10 in univariate analysis were entered into the multivariable logistic regression model.

Receiver operating characteristic(ROC) curve was constructed to measure the predictive value of serum Hcy and hsCRP for ENI. The cut-off point for each variable was assessed by Youden index. *P* value < 0.05 was considered statistically significant.

### Inclusion and exclusion criteria

**Fig. 1:**
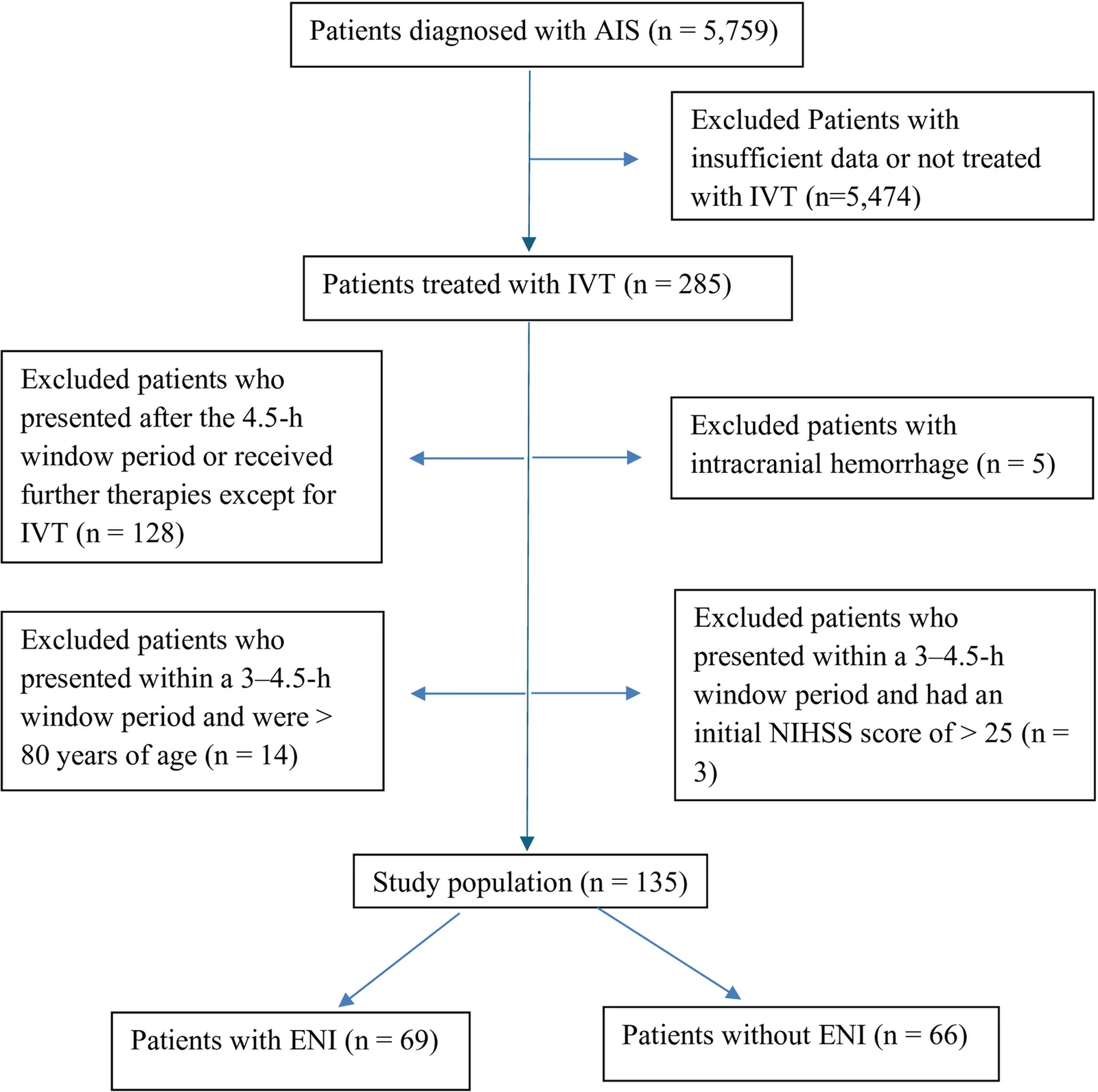
Flow chart of the study population. *Abbreviations:* AIS, acute ischemic stroke; ENI, early neurological improvement; IVT, intravenous thrombolysis; NIHSS, National Institutes of Health Stroke Scale

The inclusion criteria were (i) diagnosis of AIS confirmed within 4.5 h of symptom onset, with initiation of tPA treatment, and (ii) age > 18 years. The exclusion criteria were (i) history of further therapies except for IVT, (ii) ongoing intracranial hemorrhage, (iii) a history of a bleeding disorder, and (iv) incomplete clinical data. Additional exclusion criteria during the 3–4.5-h window period were (i) age > 80 years, (ii) severe stroke (National Institutes of Health Stroke Scale [NIHSS] score > 25), (iii) history of diabetes mellitus (DM) or stroke, and (iv) oral anticoagulant therapy, regardless of the prothrombin time international normalized ratio [15]. The eligibility criteria for tPA therapy were (i) an initial NIHSS score ≥ 4 or disabling stroke and (ii) the absence of any definite exclusion criteria for tPA therapy. Patients who underwent additional reperfusion procedures beyond IVT (e.g., mechanical thrombectomy or intra-arterial thrombolysis) were excluded to minimize confounding from post-IVT treatment effects. In the registry extract, late-window presentation and additional reperfusion therapy were aggregated as a single exclusion category (*n* = 128). Figure 1 presents a flowchart of the study population.

## Results

### General patient characteristics

This retrospective study was conducted in accordance with the tenets of the 1964 Declaration of Helsinki. The study was approved by the institutional review board of Chung-Ang University Hospital, which waived the requirement for informed consent. However, all patients whose data were retrospectively analyzed were informed of the use of their anonymized clinical information and were offered the opportunity to object to its use or publication. Patients were enrolled between October 2008 and September 2023 at a single stroke center (Department of Neurology, Chung-Ang University Hospital, Chung-Ang University School of Medicine). Patients and family members provided informed consent for IVT.

**Fig. 2:**
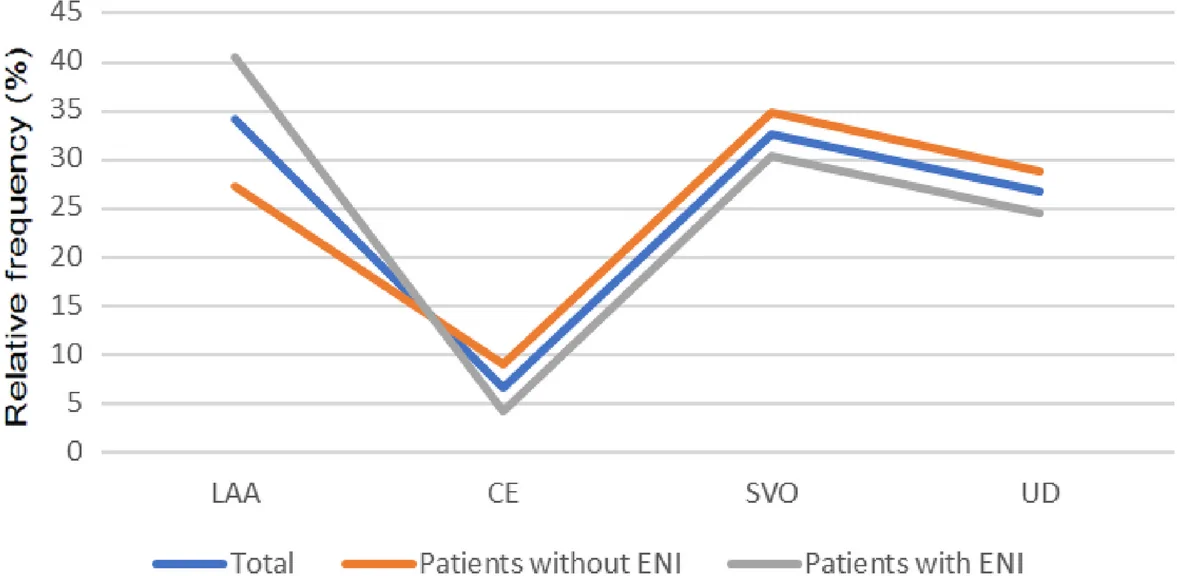
TOAST distribution of the study population. *Abbreviations:* CE, cardioembolism; ENI, early neurological improvement; LAA, large artery atherosclerosis; SVO, small vessel occlusion; TOAST, Trial of Org 10,172 in Acute Stroke Treatment; UD, undetermined

The study included 135 patients with AIS eligible for tPA treatment. Among them, 69 patients (51.1%) had good outcomes (ENI). The mean age of the patients was 71.00 ± 12.95 years. Regarding the risk factors for stroke, 58.5% of the included patients had hypertension, 31.9% had atrial fibrillation, 22.2% had a habit of smoking, and 17.8% had a history of stroke. Patients without ENI (*n* = 66) had higher mean serum Hcy, hsCRP, serum low-density lipoprotein cholesterol, triglyceride, and creatinine levels than those with ENI. The baseline clinical findings and demographic characteristics are summarized in Table 1, and Fig. 2. Although not statistically significant, LAA was numerically more frequent in the ENI group than in the non-ENI group, suggesting a possible trend toward better IVT response in atherosclerotic stroke mechanisms.

**Table 1:** Baseline clinical and demographic characteristics of the included patients

	Patients (*n* = 135)	Patients without ENI (*n* = 66)	Patients with ENI (*n* = 69)	P-value
Age, years	71.00 ± 12.95	69.00 ± 11.43	74.00 ± 13.92	0.065
Gender, male	90 (66.7%)	45 (68.2%)	45 (65.2%)	0.201
Underlying disease				
- HTN	79 (58.5%)	34 (51.5%)	45 (65.2%)	0.056
- DM	27 (20.0%)	14 (21.2%)	13 (18.8%)	0.052
- A.fib	43 (31.9%)	22 (33.3%)	21(30.4%)	0.467
- IHD	18 (13.3%)	9 (13.6%)	9 (13.0%)	0.207
- History of stroke	24 (17.8%)	13 (19.7%)	11 (15.9%)	1.000
Current smoking	30 (22.2%)	17 (25.8%)	13 (18.8%)	0.680
TOAST				0.738
- LAA	46 (34.1%)	18 (27.3%)	28 (40.6%)	
- CE	9 (6.7%)	6 (9.1%)	3 (4.3%)	
- SVO	44 (32.6%)	23 (34.8%)	21 (30.4%)	
- UD	36 (26.7%)	19 (28.8%)	17 (24.6%)	
Initial NIHSS	8.00 ± 5.44	8.00 ± 5.76	7.00 ± 5.11	0.894
- mild stroke(≤ 5)	55(40.7%)	25(37.9%)	30(43.5%)	
- moderate to severe stroke(> 6)	80(59.3%)	41(62.1%)	39(56.5%)	
Initial SBP (mmHg)	150.00 ± 28.29	150.00 ± 29.56	150.00 ± 27.23	0.094
Initial DBP (mmHg)	85.00 ± 15.18	83.00 ± 14.92	89.00 ± 15.54	0.361
Hcy (umol/L)	12.38 ± 7.52	13.57 ± 9.05	11.29 ± 5.24	0.003
hsCRP (mg/L)	1.11 ± 3.02	1.15 ± 3.19	1.10 ± 2.86	< 0.001
Hb (g/dl)	14.10 ± 1.61	13.80 ± 1.54	14.40 ± 1.69	0.304
Plt (K)	214.00 ± 48.36	216.00 ± 43.40	213.00 ± 52.89	0.710
LDL-C (mg/dL)	109.00 ± 39.10	110.00 ± 39.69	109.00 ± 38.66	0.009
TG (mg/dL)	100.00 ± 164.87	134.00 ± 169.93	104.00 ± 161.13	0.001
Cr (mg/dL)	0.93 ± 0.50	0.97 ± 0.53	0.90 ± 0.47	0.094
HbA1c (%)	5.70 ± 1.31	5.80 ± 1.37	5.65 ± 1.26	0.141
PT (INR)	1.00 ± 2.24	1.01 ± 0.08	0.99 ± 3.13	0.062

Quantitative data were presented as mean ± standard deviation values, while categorical variables were expressed as absolute values (percentages)

*Abbreviations*: *A.fib* atrial fibrillation, *CE* cardioembolism, *Cr* creatinine, *DBP* diastolic blood pressure, *DM* diabetes mellitus, *ENI* early neurological improvement, *Hb* hemoglobin, *HbA1c* hemoglobin A1c, *Hcy* homocysteine, *hsCRP* high-sensitivity C-reactive protein, *HTN* hypertension, *IHD* ischemic heart disease, *LAA* large artery atherosclerosis, *LDL-C* low density lipoprotein cholesterol, *NIHSS* National Institutes of Health Stroke Scale, *Plt* platelet, *PT (INR)* prothrombin time (international normalized ratio), *SBP* systolic blood pressure, *SVO* small vessel occlusion, *TG* triglyceride, *TOAST* Trial of Org 10,172 in Acute Stroke Treatment, *UD* undetermined

### Binary logistic regression analysis

**Fig. 3:**
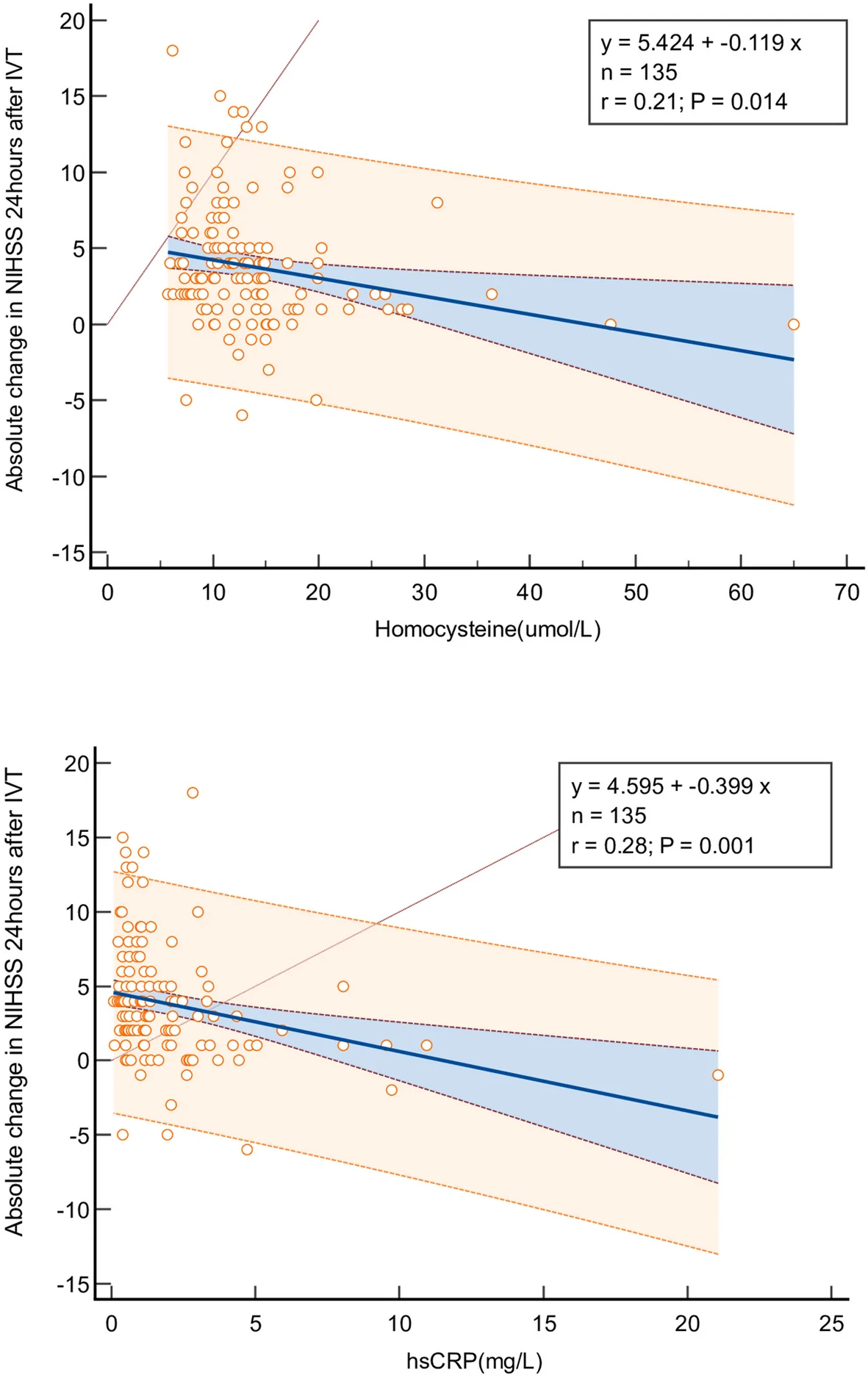
Association between serum Hcy, hsCRP, and absolute change in NIHSS 24 h after IVT. *Abbreviations*: Hcy, homocysteine; hsCRP, high-sensitivity C-reactive protein; IVT, intravenous thrombolysis; NIHSS, National Institutes of Health Stroke Scale

A binary logistic regression model was used to identify statistically significant factors affecting ENI. In univariate analysis, significant factors were age(OR, 0.996; 95% CI, 0.938–0.994; *P = *0.017), underlying hypertension(OR, 2.000; 95% CI, 0.998–4.008; *P = *0.051), diabetes mellitus(OR, 0.400; 95% CI, 0.165–0.970; *P = *0.043), TOAST-SVO(OR, 1.853; 95% CI, 0.890–3.859; *P = *0.099), Hcy level (OR, 0.829; 95% CI, 0.754–0.911; *P < *0.001), hsCRP(OR, 0.451; 95% CI, 0.306–0.662; *P < *0.001), LDL-C(OR, 0.983; 95% CI, 0.974–0.993; *P = *0.001), and TG(OR, 0.997; 95% CI, 0.994–1.000; *P = *0.070). Initial SBP and DBP were not included in the regression analyses, as blood pressure status was captured by the hypertension variable to avoid multicollinearity. Multivariate logistic regression analysis was performed on the factors that yielded significant results in the univariate logistic regression analysis. Variables with P ≤ 0.10 in univariate analysis were entered into the multivariable logistic regression model. TOAST-SVO, TG, and LDL-C met the *P < *0.10 threshold in univariate analysis but were not retained in the final multivariable model following stepwise selection, as their associations were attenuated after adjustment for other covariates. Young age (OR, 0.958; 95% CI, 0.922–0.996; *P = *0.029), absence of DM (OR, 0.212; 95% CI, 0.055–0.819; *P = *0.024), low serum Hcy level (OR, 0.865; 95% CI, 0.787–0.950; *P = *0.003), and hsCRP level (OR, 0.345; 95% CI, 0.203–0.586; *P < *0.001) were significantly associated with a high incidence of ENI after tPA therapy. Underlying HTN (OR, 6.139; 95% CI, 2.016–18.698; *P = *0.001) was associated with ENI. (Table 2). Furthermore, serum Hcy and hsCRP were also related to absolute change in NIHSS 24 h after IVT. Figure 3 presents scatter diagram and regression line. The lower the initial values of serum Hcy and hsCRP, the greater the change in NIHSS scores 24 h after tPA.

**Table 2:** Factors affecting ENI after IVT using binary logistic regression models

	Univariate analysis			Multivariate analysis		
	P-value	Odds ratio	95% CI	P-value	Odds ratio	95% CI
Age, years	0.017	0.966	0.938–0.994	0.029	0.958	0.922–0.996
Gender, male	0.146	1.711	0.830–3.525			
Underlying disease						
- HTN	0.050	2.000	0.998–4.008	0.001	6.139	2.016–18.698
- DM	0.043	0.400	0.165–0.970	0.024	0.212	0.055–0.819
- A.fib	0.455	1.319	0.637–2.731			
- IHD	0.163	2.105	0.740–5.985			
- History of stroke	0.904	0.947	0.392–2.290			
Current smoking	0.581	0.795	0.353–1.794			
TOAST						
- LAA	0.203	0.628	0.307–1.286			
- CE	0.680	0.751	0.193–2.926			
- SVO	0.099	1.853	0.890–3.859			
- UD	0.351	0.694	0.322–1.495			
Initial NIHSS	0.653	0.986	0.926–1.049			
Hcy (umol/L)	< 0.001	0.829	0.754–0.911	0.003	0.865	0.787–0.950
hsCRP (mg/L)	< 0.001	0.451	0.306–0.662	< 0.001	0.345	0.203–0.586
LDL-C (mg/dL)	0.001	0.983	0.974–0.993	0.093	0.988	0.975–1.002
TG (mg/dL)	0.070	0.997	0.994–1.000			
Cr (mg/dL)	0.199	0.612	0.289–1.295			
PT (INR)	0.592	0.853	0.477–1.525			

*Abbreviations*: *A.fib* atrial fibrillation, *CE* cardioembolism, *CI* confidence intervals, *Cr* creatinine, *DBP* diastolic blood pressure, *DM* diabetes mellitus, *Hb* hemoglobin, *HbA1c* hemoglobin A1c, *Hcy* homocysteine, *hsCRP* high-sensitivity C-reactive protein, *HTN* hypertension, *IHD* ischemic heart disease, *LAA* large artery atherosclerosis, *LDL-C* low density lipoprotein cholesterol, *NIHSS* National Institutes of Health Stroke Scale, *Plt* platelet; PT (INR), prothrombin time (international normalized ratio), *TG* triglyceride, *SBP* systolic blood pressure, *SVO* small vessel occlusion, *TOAST* Trial of Org 10,172 in Acute Stroke Treatment, *UD* undetermined

### ROC curve and cut-off values associated with ENI

**Fig. 4:**
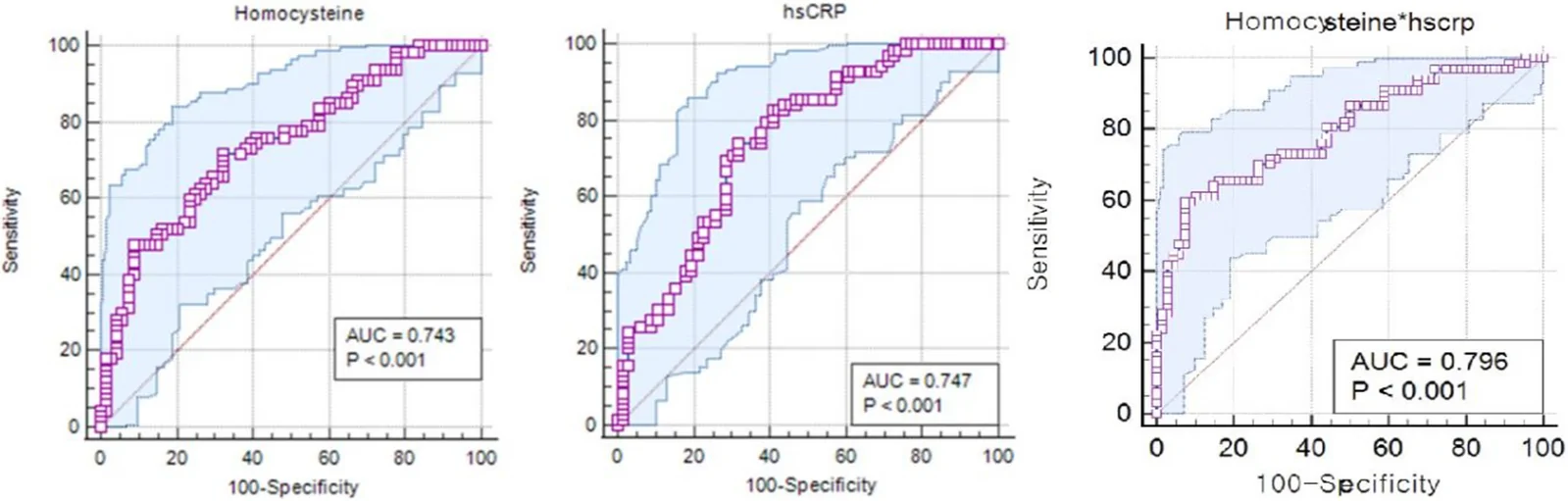
Receiver-operating characteristic (ROC) curves of serum homocysteine and high-sensitivity C-reactive protein (hsCRP) for early neurological improvement. *Abbreviations:* Hcy, homocysteine; hsCRP, high-sensitivity C-reactive protein; ENI, early neurological improvement; ROC, receiver operating characteristic

As shown Table 3 and Fig. 4, ROC curve and optimal cut-off values of serum Hcy and hsCRP for predicting ENI were measured. Areas under ROC curve(AUC) and cut-off points of serum Hcy and hsCRP were 0.743, 11.99 umol/L, 0.747, and 1.16 mg/L, respectively. Sensitivity and specificity of Hcy were 68.1 and 72.7%. In addition, sensitivity and specificity of hsCRP were 73.9 and 68.2%. If Hcy and hsCRP were combined and used together, the ROC was 0.797, which predicted ENI better than when Hcy and hsCRP was used alone. (sensitivity 92.75, specificity 60.61, and optimal cut-off value 23.65).

**Table 3:** Cut-off values of Hcy and hsCRP associated with ENI

	Areas under ROC curve	95% CI	*P*-value	Optimal cut-off value
Hcy(umol/L)	0.743	0.660–0.814	< 0.001	11.99
hsCRP(mg/L)	0.747	0.665–0.818	< 0.001	1.16
Hcy*hsCRP	0.797	0.719–0.861	< 0.001	23.65

*Abbreviations*: *CI* confidence intervals, *ENI* early neurological improvement, *Hcy* homocysteine, *hsCRP* high-sensitivity C-reactive protein, *ROC* receiver operating characteristic

In ROC analyses (ENI coded as the positive state), homocysteine and hsCRP each showed modest discrimination (AUC 0.743 and 0.747, respectively), and the combined marker model improved discrimination (AUC 0.797). The Youden index identified thresholds of 11.99 μmol/L (homocysteine) and 1.16 mg/L (hsCRP). Because both biomarkers were inversely associated with ENI in multivariable models, the corresponding decision inequalities are Hcy ≤ 11.99 μmol/L and hsCRP ≤ 1.16 mg/L when predicting ENI; values above these thresholds align with a lower ENI probability. These thresholds are data-driven decision points for discrimination in this cohort and are not clinical ‘normal’ limits. In addition, these findings indicate association and discrimination rather than causation; we did not compare categorical ‘mild/moderate’ elevations versus ‘normal’, and our interpretation avoids categorical prognostication beyond the presented analyses.

## Discussion

We investigated factors affecting treatment outcomes in patients with AIS who received tPA therapy. The results showed that age, serum Hcy and hsCRP levels, HTN, and DM were associated with ENI.

Among the mechanisms of AIS, atherosclerosis can induce vascular endothelial dysfunction and instability of lipid plaques. Previous studies reported that serum Hcy and hsCRP levels are associated with atherosclerosis [16]. Hyperhomocysteinemia increases the risk of cerebral stroke, potentially owing to vascular atherosclerotic modifications [17]. Hcy promotes the production of nitrogen oxide, which causes overgrowth of vascular smooth muscles, eventually leading to the dilatation of blood vessels and accumulation of cholesterol [18, 19]. hsCRP is the most sensitive indicator of atherosclerosis and increases rapidly during ischemic stroke, initiating lipid accumulation in vascular endothelial cells [20, 21]. This activates the production of chemicals that attract monocytes and may increase plasma thrombin levels, which has a significant impact on the formation of blood clots [22].

Epidemiological research has been conducted on the roles of Hcy and hsCRP. Several studies suggested that serum Hcy levels are related to functional disability in the acute phase of stroke [23–25]. Elevated serum hsCRP level has also been suggested to be a risk factor for poor outcomes in patients with AIS. Some studies reported that the median serum hsCRP level was higher in the unfavorable functional outcome group than in the favorable functional outcome group [26–28]. However, the impact of Hcy and hsCRP on ENI has not been closely studied until now. In this study, we presented cut-off points for Hcy and hsCRP that help predict ENI. Furthermore, we confirmed that using Hcy and hsCRP simultaneously is better for understanding ENI than only using Hcy or hsCRP. This can be seen as a difference from other studies.

We excluded patients who experienced further therapies except for IVT. If we chose patients who underwent treatments other than tPA, the success rate of the procedure itself could have influenced neurological functions apart from any other risk factor [29, 30]. In other words, if the initial NIHSS score is high enough to undergo mechanical thrombectomy or other surgical treatments, the patient’s neurological prognosis worsens, regardless of serum Hcy and hsCRP levels.

Patients with mild or moderate cardioembolic stroke may benefit more from direct mechanical thrombectomy than bridging therapy [31]. Since our study excluded patients who received further treatment, the proportion of patients with cardioembolic stroke was low. The rate of ENI after IVT in patients with cardioembolic stroke was inevitably low.

Building on these mechanistic considerations, lower concentrations of homocysteine and hsCRP were associated with a higher probability of ENI, whereas higher concentrations were associated with a lower probability of ENI. In multivariable analyses, younger age and the absence of diabetes mellitus were similarly associated with higher ENI probability. Hypertension showed a positive association with ENI (OR 6.139), though the underlying mechanism is not fully understood and may be confounded by other clinical factors. This finding warrants further investigation. In ROC analyses, homocysteine and hsCRP each demonstrated moderate discrimination, and a combined marker model modestly improved discrimination (AUC 0.797 vs. 0.743 and 0.747, respectively). The resulting thresholds represent data‑driven operating points that maximize sensitivity and specificity in this dataset and are not intended as pathophysiologic cut‑points; prospective validation is warranted.

Beyond statistical association, these biomarker findings may help contextualize early prognostic assessment after IVT when interpreted alongside clinical factors; external validation and clinical impact evaluation should be assessed.

The study has some limitations. First, this was a single-center, retrospective study with a small sample size. We need to increase the number of participating institutions and patients to obtain more generalizable results. Second, the present study was designed to determine the effects of serum Hcy and hsCRP levels on the prognosis of patients who received IVT. Therefore, we did not include patients who did not undergo thrombolysis. This may have induced selection bias. In addition, late‑window presentation and receipt of further reperfusion therapy were aggregated as a single exclusion category in the registry extract (*n* = 128), precluding finer stratification; this represents a limitation of the retrospective dataset. Third, serum Hcy levels may depend on factors such as genetics and sex [32]. We did not perform a real-time evaluation of these factors; this was difficult to do so due to the retrospective nature of the study. A more extensive prospective study must be conducted to address these limitations. Finally, because only patients who met strict eligibility criteria for IVT with available biomarker data were included, the analyzed cohort represented a relatively small proportion of the overall AIS population treated at our center, which may limit the generalizability of the findings. Long-term functional outcomes such as the modified Rankin Scale were not analyzed because consistent follow-up data were not available in this retrospective dataset.

## Conclusion

In patients with acute ischemic stroke treated with intravenous alteplase, lower homocysteine and lower hsCRP were independently associated with a higher probability of early neurological improvement. The combined marker approach showed improved discrimination compared with either marker alone and may help contextualize early prognostic assessment after IVT; further validation should be assessed.

## Data Availability

The data supporting the findings of this study are available in the supplementary materials. Further inquiries can be directed to the authors.
